# Mitochondrial Haplogroups and Left Ventricular Diastolic Dysfunction in People Living With and Without HIV

**DOI:** 10.1093/infdis/jiag090

**Published:** 2026-02-11

**Authors:** Craig Cronin, Jing Sun, Jorge R Kizer, Katherine C Wu, Wendy S Post, David C Samuels, Todd Hulgan, Brad Aouizerat, Frank Palella, Shehnaz Hussain, Jeremy Martinson, Nicole D Armstrong, Claudia Martinez, Caitlin A Moran, Alan Hinderliter, Yasmeen Golzar, Federico M Asch, Jason Lazar, Carlos J Rodríguez, Todd T Brown

**Affiliations:** Department of Medicine, Johns Hopkins University, Baltimore, Maryland, USA; Department of Epidemiology, Johns Hopkins Bloomberg School of Public Health University, Baltimore, Maryland, USA; Department of Medicine (Cardiology), San Francisco Veterans Affairs Health Care System and University of California, San Francisco, San Francisco, California, USA; Department of Epidemiology and Biostatistics, University of California, San Francisco, San Francisco, California, USA; Department of Medicine (Cardiology), Johns Hopkins University, Baltimore, Maryland, USA; Department of Epidemiology, Johns Hopkins Bloomberg School of Public Health University, Baltimore, Maryland, USA; Department of Medicine (Cardiology), Johns Hopkins University, Baltimore, Maryland, USA; Department of Molecular Physiology and Biophysics, Vanderbilt School of Medicine, Nashville, Tennessee, USA; Department of Medicine (Infectious Diseases), Vanderbilt University Medical Center, Nashville, Tennessee, USA; Department of Oral and Maxillofacial Surgery, New York University, New York, New York, USA; Translational Research Center, College of Dentistry, New York University, New York, New York, USA; Department of Medicine (Infectious Diseases), Northwestern University, Chicago, Illinois, USA; Department of Public Health Sciences, School of Medicine and Comprehensive Cancer Center, University of California, Davis, Davis, California, USA; Department of Infectious Diseases and Microbiology, School of Public Health, University of Pittsburgh, Pittsburgh, Pennsylvania, USA; Department of Epidemiology, University of Alabama at Birmingham, Birmingham, Alabama, USA; Department of Medicine (Cardiology), University of Miami Miller School of Medicine, Miami, Florida, USA; Department of Medicine (Infectious Diseases), Emory University School of Medicine, Atlanta, Georgia, USA; Department of Medicine (Cardiology), University of North Carolina at Chapel Hill, Chapel Hill, North Carolina, USA; Department of Medicine (Cardiology), Cook County Health, Chicago, Illinois, USA; Department of Medicine (Cardiology), MedStar Health Research Institute, MedStar Washington Hospital Center, Washington, DC, USA; Department of Medicine, Georgetown University School of Medicine, Washington, DC, USA; Department of Medicine (Cardiology), SUNY Downstate Health Sciences University, Brooklyn, New York, USA; Department of Medicine (Cardiology), Albert Einstein College of Medicine, Bronx, New York, USA; Department of Epidemiology, Albert Einstein College of Medicine, Bronx, New York, USA; Department of Epidemiology, Johns Hopkins Bloomberg School of Public Health University, Baltimore, Maryland, USA; Department of Medicine (Endocrinology), Johns Hopkins University, Baltimore, Maryland, USA

**Keywords:** mitochondrial genetics, cardiac dysfunction, left ventricular diastolic dysfunction, HIV, mitochondrial-toxic antiretroviral therapy

## Abstract

**Background:**

Cardiac dysfunction is more common in people with HIV (PWH) than those without HIV (PWoH), with mitochondrial dysfunction implicated in pathogenesis. We investigated whether variations in mitochondrial DNA (mtDNA) and certain dideoxynucleoside analogs (D-drugs) relate to left ventricular diastolic dysfunction (LVDD) in PWH.

**Methods:**

We included individuals with echocardiograms from the Multicenter AIDS Cohort Study and Women's Interagency HIV Study. LVDD was defined using characterizing heart function on antiretroviral therapy criteria. mtDNA haplogroups were inferred using HaploGrep. Separate exploratory multivariable logistic regressions examined associations between LVDD and African (L0L1, L2, L3, or “other”) or European haplogroups (UK, H, JT, or “other”), D-drugs, and their interactions. No adjustments were made for multiple comparisons.

**Results:**

Among 842 men (455 PWH and 387 PWoH) and 898 women (620 PWH and 278 PWoH), LVDD prevalence was 29% in women and 24% in men. Among non-Hispanic White men with HIV, European haplogroup H was associated with lower odds of LVDD (odds ratio [OR], 0.50; 95% CI, 0.26–0.93), while haplogroup clade JT was associated with increased odds (OR, 2.09; 95% CI, 1.00–4.36). In men with HIV, D-drug exposure was associated with increased odds of LVDD (OR, 1.94; 95% CI, 1.21–3.13). No significant associations were observed between haplogroups and LVDD in women. HIV serostatus modified the association of haplogroup L2 (*p*_interaction_ = 0.036) and L3 (*p*_interaction_ = 0.045) with LVDD in women.

**Conclusions:**

Mitochondrial genetic variation and D-drug use were associated with altered LVDD risk in men with HIV, highlighting potential biological mechanisms that may be targeted for surveillance or therapeutic strategies.

## Teaser

Among people with HIV, certain mitochondrial DNA haplogroups were associated with altered susceptibility to left ventricular diastolic dysfunction. Additionally, prior exposure to mitochondrially toxic D-drugs were associated with increased odds of left ventricular diastolic dysfunction.

As the life expectancy of people with HIV (PWH) continues to increase, certain comorbidities, such as cardiovascular disease (CVD), have become more prevalent and now represent a leading cause of death in this population [1–3]. Cardiac dysfunction occurs at a higher incidence and prevalence among PWH and contributes significantly to morbidity and mortality, accounting for nearly one-quarter of deaths [4, 5]. Given the heart's high energy demands, mitochondrial metabolic alterations are believed to play a central role in the origin and progression of cardiac dysfunction, including in the development of heart failure (HF) [6–10].

In PWH, HIV infection is known to cause mitochondrial dysfunction, driven in part by chronic inflammation and oxidative stress [11–13]. Additionally, exposure to certain antiretroviral therapies (ART), such as nucleoside reverse transcriptase inhibitors, can cause mitochondrial dysfunction [14, 15]. Specifically, dideoxynucleoside analogs (D-drugs), such as stavudine (D4T), zalcitabine (ddC), and didanosine (ddI) inhibit the mitochondrial specific DNA polymerase (DNA pol-γ) causing impaired mtDNA replication and function, with dysfunction persisting even after DNA pol-γ recovers following discontinuations of these therapies [15, 16]. Therefore, people with chronic HIV infection are susceptible to mitochondrial damage through multiple factors.

Mitochondrial function is related to mitochondrial DNA (mtDNA) variation, which can be summarized using mtDNA haplogroups. These mtDNA haplogroups, which constitute unique clusters of single nucleotide polymorphisms (SNPs) that influence mitochondrial processes and trace each individual's prehistoric ancestry, have been independently associated with several diseases, including CVD and diabetes [17–22]. However, few studies have examined the relationship between mtDNA haplogroups and cardiac dysfunction in the general population, and those that have were primarily focused on ischemic heart disease and later-stage ischemic cardiomyopathy [23, 24]. To our knowledge, no studies have analyzed associations between mtDNA haplogroups in PWH and left ventricular diastolic dysfunction (LVDD), which can precede and increase the risk for HF, especially HF with preserved ejection fraction.

To address this gap, we aimed to evaluate the association between mtDNA haplogroups and LVDD across the entire study population and separately by HIV serostatus. Among PWH, we also assessed the potential impact of prior D-drug exposure on LVDD, with the goal of identifying additional contributors to mitochondrial dysfunction beyond HIV infection alone.

## METHODS

### Study Population

The Multicenter AIDS Cohort Study (MACS) and the Women's Interagency HIV Study (WIHS) were large-scale, prospective observational cohort studies designed to evaluate the impact of HIV infection among men and women across multiple centers. Both cohorts include individuals with and without HIV who share similar sociodemographic and behavioral characteristics. The institutional review board at each WIHS and MACS site listed in the Supplementary Materials approved the study protocol with each participant providing written informed consent.

Participants were enrolled during 4 time periods for WIHS (1994–95, 2001–02, 2011–12, and 2013–15) and 3 for MACS (1984–85, 1987–90, and 2010–18). Participants attended semiannual visits with standardized interviews, physical examinations, and laboratory testing. Detailed descriptions of the study design, enrollment, and data collection are reported elsewhere [25–28].

For the current analysis, WIHS participants were included if they self-reported being non-Hispanic Black, had available genotyping data, and participated in the WIHS-wide echocardiographic study [29]. Due to few participants of European ancestry in WIHS, the WIHS analyses were restricted to individuals of African ancestry. MACS participants were included if they self-reported being non-Hispanic Black or non-Hispanic White, had available genotyping data, and participated in the MACS-wide echocardiographic study [30]. MACS and WIHS participants who self-reported as Hispanic were excluded from the analysis due to limited sample size.

Additional information regarding the MACS and WIHS cross-sectional echocardiographic studies are described elsewhere with a brief discussion in the Supplementary Materials [29, 30].

### Assessment of Left Ventricular Diastolic Dysfunction

All scans for each echocardiographic study were analyzed in a central core lab. The primary outcome was isolated LVDD (in the absence of LV systolic dysfunction). LV systolic dysfunction was not evaluated because of its low prevalence [29, 30]. LVDD was defined using criteria from the Characterizing Heart Function on Antiretroviral Therapy (CHART) study, which was specifically developed for HIV, and yield higher prevalence of LVDD than the American Society of Echocardiography (ASE) criteria [30, 31]. Individual echocardiographic parameters are defined elsewhere and briefly described in the Supplementary Materials [30, 31].

### Mitochondrial DNA Haplogroup Classification

SNPs in the mtDNA were used to group participants into common African (L0L1, L2, L3, or “other”) and European (UK, H, JT, and “other”) haplogroups or clades using HaploGrep. Additional details regarding the process used for mtDNA haplogroup determination are described extensively elsewhere [17, 21]. The MACS analysis included individuals of both African and European mitochondrial haplogroups, while the WIHS cohort was restricted to African haplogroups.

### Covariate Definitions

The HIV serostatus in people without HIV (PWoH) was assessed annually to confirm seronegativity. Among PWH, measures of HIV disease activity included current and nadir CD4+ T lymphocyte cell count, CD4/CD8 ratio, history of clinical AIDS, and plasma HIV RNA concentration. ART exposure was self-reported at each visit. D-drug exposure was defined based on self-report as ever previous exposure to stavudine, zalcitabine, or didanosine prior to the echocardiogram and summarized as a binary variable.

Sociodemographic and behavioral variables included age, self-reported race, study site, highest educational attainment, and self-reported substance use. Smoking status was categorized as never or current/former smoker. Alcohol use was defined based on reported use since the previous visit. Injection and noninjection drug use (heroin or cocaine) were captured as binary variables indicating any prior use. Additional covariates included principal components of nuclear genetic ancestry [21], hepatitis C virus (HCV) seropositivity, and body mass index (BMI) determined from standardized measurement of height and weight. Hypertension (HTN) was defined as systolic blood pressure ≥130, diastolic blood pressure ≥80, or use of HTN medications with ever self-reported diagnosis. Dyslipidemia was defined as total cholesterol ≥200 mg/dL, fasting LDL ≥130 mg/dL, HDL <40 mg/dL, or use of lipid lowering medication with self-report or clinical diagnosis in the past. Diabetes mellitus (DM) and estimated glomerular filtration rate (eGFR) were defined or calculated based upon previous definitions or validated formulas [32, 33]. All covariates, except for injection and noninjection drug use, were determined using the most recent information available at or prior to the echocardiogram.

### Statistical Analyses

All statistical analyses were conducted using R version 2024.01. Multivariable logistic regression models were used to examine the sex- and race-stratified association between African haplogroups (L0L1, L2, L3, or “other”) for males and females or European haplogroups (H, UK, JT, or “other”) for males and LVDD.

Following univariable analyses examining the associations between mtDNA haplogroups and LVDD without covariate adjustment, 3 sequential multivariable models were constructed. Model 1 adjusted for 2 principal components of nuclear genetic ancestry, age, and site of enrollment. Model 2 further adjusted for educational attainment, BMI, smoking, alcohol use, history of noninjection or injection drug use, and HCV serostatus. Model 3 additionally included clinical risk factors relevant to LVDD, namely, HTN, DM, dyslipidemia, and eGFR. Interaction terms between HIV serostatus and mtDNA haplogroups were added to Model 3 without stratification by HIV serostatus.

Sensitivity analyses were conducted among PWH using model 3 covariates with the addition of HIV-related disease severity measures, including CD4/CD8 ratio, history of clinical AIDS, nadir CD4 count, and 3-year average viremia. A 3-year average viremia was used given its superior utility compared with single time-point viral load measurements [34]. A separate sensitivity analysis was restricted to PWH with plasma HIV RNA concentration ≤20 copies/mL.

In analyses restricted to PWH assessing the relationship between D-drug exposure and LVDD, models were stratified by sex and adjusted for HIV-specific covariates, including CD4/CD8 ratio, history of clinical AIDS, nadir CD4 count, and 3-year average viremia. An additional analysis using covariates from model 3 and HIV-specific variables included an interaction term between D-drug exposure and mtDNA haplogroups. Further stratified analyses by haplogroup or D-drug exposure were performed to better characterize the independent and joint effects of these measures on LVDD. Covariate selection for all models was guided by prior literature on mitochondrial haplogroups and cardiovascular outcomes, as well as findings from preliminary univariable analyses [17, 21, 29, 30].

## RESULTS

Participant characteristics stratified by HIV serostatus and sex are presented in Table 1. Among the 898 non-Hispanic Black women from the WIHS cohort, the median age was 52 years (IQR: 45, 57), with 69% being PWH. The most common mitochondrial haplogroups among these women were L3 (41%) and L2 (29%) (Table 2). Overall, women with HIV were more likely to have a history of HCV infection, HTN, and dyslipidemia, but were less likely to report smoking, alcohol use since their last visit, or drug use compared with women without HIV.

**Table 1.** Characteristics of Study Participants by Sex and HIV Serostatus

	Male		Female	
	HIV− (n = 387)	HIV + (n = 455)	HIV− (n = 278)	HIV + (n = 620)
Race				
Black	89 (23.0)	179 (39.3)	278 (100)	620 (100)
White	298 (77.0)	276 (60.7)	0 (0)	0 (0)
Age at visit, years	63 (56, 69)	57 (50, 63)	51 (43, 58)	52 (46, 57)
BMI, kg/m^2^	27 (24, 30)	27 (24, 30)	32 (26, 38)	32 (26, 38)
Education, n (%)				
Less than high school	16 (4.1)	34 (7.5)	87 (31.3)	210 (33.9)
Completed high school	41 (10.6)	85 (18.7)	94 (33.8)	205 (33.1)
Completed at least 4-year college degree	330 (85.3)	336 (73.8)	97 (34.9)	205 (33.1)
Smoking status, n (%)				
Former or Current	252 (65.1)	316 (69.4)	204 (73.4)	419 (67.6)
Never	135 (34.9)	139 (30.6)	74 (26.6)	201 (32.4)
Alcohol intake, n (%)				
Alcohol since last visit	315 (81.4)	336 (73.8)	164 (59.0)	270 (43.5)
No alcohol since last visit	72 (18.6)	119 (26.2)	114 (41.0)	350 (56.5)
Noninjection drug use, n (%)				
Yes	158 (40.8)	249 (54.7)	120 (43.2)	173 (27.9)
Injection drug use, n (%)				
Yes	27 (7.0)	69 (15.2)	18 (6.5)	18 (2.9)
HCV, n (%)				
Yes	27 (7.0)	58 (12.8)	37 (13.3)	112 (18.1)
HTN, n (%)				
Yes	289 (74.7)	337 (74.1)	195 (70.1)	467 (75.3)
Use of blood pressure medication	177 (45.7)	191 (42.0)	134 (48.2)	330 (53.2)
Diabetes, n (%)				
Yes	83 (21.5)	107 (23.5)	63 (22.7)	132 (21.3)
Dyslipidemia, n (%)				
Yes	284 (73.4)	328 (72.1)	144 (51.8)	405 (65.3)
Use of lipid lowering medication	172 (44.4)	196 (43.1)	58 (20.9)	141 (22.7)
eGFR	84 (73, 94)	81 (66, 95)	98 (84, 113)	90 (72, 108)
LVDD chart, n (%)				
Yes	94 (24.3)	110 (24.2)	89 (32.0)	175 (28.2)
HIV disease severity				
CD4+ count, cells/mm^3^	–	701 (497, 906)	–	696 (502, 906)
CD4/CD8 ratio	1.8 (1.3, 2.4)	0.9 (0.7, 1.3)	2.2 (1.7, 2.8)	0.9 (0.6, 1.3)
Viral load undetectable (<20 copies/mL), n (%)	…	355 (78.0)	…	453 (73.0)
3-y Average HIV viremia (copies/mL)^a^	…	16.8 (10.8, 54.4)	…	15.8 (4.2, 261.9)
History of AIDS, n (%)				
No	–	455 (100)	–	574 (92.6)
Yes	–	0 (0)	–	46 (7.4)
Nadir CD4 before ART, cells/mm^3^	…	311 (188–460)	…	290 (157–430)
ART therapy, n (%)				
ART at echo				
No	–	20 (4.4)	–	48 (7.7)
Yes, ART	–	432 (94.9)	–	571 (92.1)
Yes, monotherapy	–	3 (0.7)	–	1 (0.2)
D-drug previous use				
Yes	–	206 (45.3)	–	171 (27.6)

Values shown are median (IQR), unless otherwise indicated.

Abbreviations: AIDS, acquired immunodeficiency syndrome; ART, antiretroviral therapy; BMI, body mass index; eGFR, estimated glomerular filtration rate; HCV, hepatitis C virus; HIV, human immunodeficiency virus; HTN, hypertension; IQR, interquartile range; LVDD, left ventricular diastolic dysfunction.

^a^3-y averaged HIV viremia using annual HIV viral load measurements for 3-y prior to study visit for each participant.

**Table 2.** Mitochondrial DNA Haplogroup Frequencies by Sex and HIV Serostatus

Non-Hispanic Black—African mtDNA Haplogroups/Clades	Overall		Male		Female	
	Male (n = 268)	Female (n = 898)	HIV− (n = 89)	HIV + (n = 179)	HIV− (n = 278)	HIV + (n = 620)
L0L1	56 (20.9)	208 (23.2)	20 (22.5)	36 (20.1)	60 (21.6)	148 (23.9)
L2	89 (33.2)	257 (28.6)	28 (31.5)	61 (34.1)	78 (28.1)	179 (28.9)
L3	105 (39.2)	366 (40.8)	35 (39.3)	70 (39.1)	112 (40.3)	254 (41.0)
Other	18 (6.7)	67 (7.5)	6 (6.7)	12 (6.7)	28 (10.1)	39 (6.3)

Non-Hispanic White—European mtDNA Haplogroups/Clades	Overall		Male		Female	
	Male (n = 574)	Female (n = 0)	HIV− (n = 298)	HIV + (n = 276)	HIV− (n = 0)	HIV + (n = 0)
UK	140 (24.4)	…	84 (28.2)	56 (20.3)	…	…
H	265 (46.2)	…	122 (40.9)	143 (51.8)	…	…
JT	122 (21.3)	…	67 (22.5)	55 (19.9)	…	…
Other	47 (8.2)	…	25 (8.4)	22 (8.0)	…	…

Values shown are n, (%).

Abbreviations: HIV, human immunodeficiency virus; mtDNA, mitochondrial DNA.

For the 842 men included from the MACS study, the median age was 59 years (IQR: 53, 66) with 54% being PWH. Most of these men identified as non-Hispanic White (68%). Among non-Hispanic Black men, L3 (39%) and L2 (33%) were the most common mitochondrial haplogroups, whereas among non-Hispanic White men, the most common haplogroups were H (46%) and UK (24%) (Table 2). Overall, men with HIV were more likely to be smokers, have a history of HCV infection, and report a history of drug use, but were less likely to have completed higher education or to have used alcohol since their last visit compared with men living without HIV.

Most women (73%) and men (78%) with HIV had undetectable plasma HIV RNA levels. Median nadir CD4 counts were 290 cells/mm^3^ (IQR: 157, 430) for women and 311 cells/mm^3^ (IQR: 187.5, 460) for men. At the time of the echocardiogram, 8% of females and 4% of males were not using ART. A higher proportion of men (45%) had a history of D-drug exposure compared with women (28%).

The prevalence of LVDD, assessed using CHART study criteria and stratified by sex and HIV serostatus, is shown in Table 1. Overall, 29% of women and 24% of men had LVDD, with women exhibiting higher rates regardless of HIV serostatus compared with men. There were no statistically significant differences in the LVDD prevalence by HIV serostatus for either men or women (*P* > .05).

**Figure 1.**
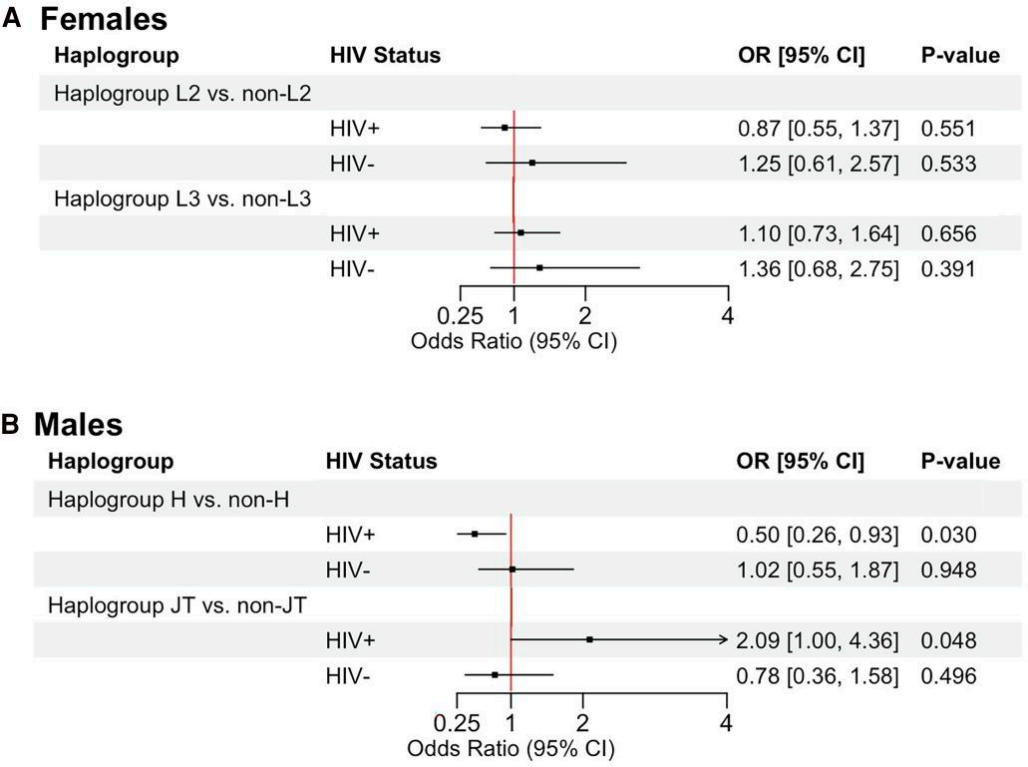
Final adjusted odds ratio using model 3 of left ventricular diastolic dysfunction (LVDD) for select mtDNA haplogroups by HIV status for males in the Multicenter AIDS Cohort Study (MACS) and females in the Women's Interagency HIV Study (WIHS). Model adjusted for 2 principal components of nuclear genetic ancestry, age, site of enrollment, educational attainment, BMI, smoking, alcohol use, history of noninjection or injection drug use, HCV serostatus, HTN, DM, dyslipidemia, and eGFR. *A*, Adjusted odds ratio of LVDD by HIV status among WIHS Participants. *B*, Adjusted odds ratio of LVDD by HIV status among MACS participants.

In a univariable logistic regression model among non-Hispanic Black women, no mtDNA haplogroup was significantly associated with LVDD. In the final adjusted model (model 3), stratified by HIV serostatus, no mtDNA haplogroup showed a significant association with LVDD among either women with or without HIV (Supplementary Table 1). Given the exploratory nature of the analysis, interactions terms were evaluated in the absence of significant main effects. HIV serostatus significantly modified the association of haplogroup L2 (*p*_interaction_ = 0.036; HIV+: OR, 0.87; 95% CI, .55–1.37; HIV−: OR, 1.25; 95% CI, .61–2.57; Figure 1*A*, Supplementary Table 1) and L3 (*p*_interaction_ = 0.045; HIV+: OR, 1.10; 95% CI, .73–1.64; HIV−: OR, 1.36; 95% CI, .68–2.75; Figure 1*A*;  Supplementary Table 1) with LVDD. Haplogroup L2 and L3 showed lower odds of LVDD among women with HIV compared with women without HIV.

Among non-Hispanic Black and non-Hispanic White men in univariable models, mtDNA haplogroups were not significantly associated with LVDD in the overall cohort not stratified by serostatus (Supplementary Table 1). In the final adjusted model restricted to PWH (model 3), European haplogroup H was associated with significantly lower odds of LVDD compared with non-H European haplogroups (OR, 0.50; 95% CI, .26–.93; Figure 1*B*), whereas European haplogroup JT was associated with a significantly higher odds of LVDD compared with non-JT haplogroups (OR, 2.09; 95% CI, 1.00–4.36; Figure 1*B*). Incorporating clinical risk factors for LVDD in model 3, compared with model 2, did not alter these associations. The results remained robust after excluding individuals with HIV RNA >20 copies/mL and after adjusting for HIV-specific variables such as CD4/CD8 ratio, history of AIDS, 3-year average viremia, and nadir CD4 count (Supplementary Tables 2 and 3). Among PWoH, no significant associations between European mtDNA haplogroups and LVDD were observed. No significant associations with LVDD were observed for African mtDNA haplogroups among men with or without HIV. Among non-Hispanic Black or White men, there were no significant interactions between HIV status and mtDNA haplogroups (Supplementary Table 1).

**Figure 2.**
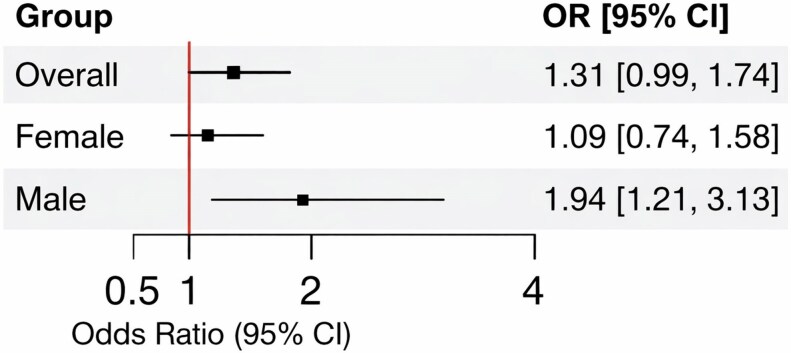
Adjusted odds ratio of left ventricular diastolic dysfunction for people with HIV and previous D-drug exposure compared with people with HIV and no previous D-drug exposure. Model adjusted for CD4/CD8 ratio, history of clinical AIDS, 3-y average HIV viremia, and nadir CD4 count.

In a univariable model including both men and women, prior D-drug exposure was associated with higher odds of LVDD (OR, 1.37; 95% CI, 1.04–1.79; Supplementary Table 4). However, the association was attenuated and became nonsignificant after adjustment for CD4/CD8 ratio, history of clinical AIDS, 3-year average viremia, and nadir CD4 count (OR, 1.31; 95% CI, .99–1.74; Figure 2). When stratified by sex using the same model, D-drug exposure was significantly associated with higher odds ratio of LVDD in men, but not in women (Figure 2). No significant interactions were observed between D-drug exposure and mtDNA haplogroups in relation to LVDD for either sex. Supplementary Tables 5 and 6 summarize the associations between D-drug exposure, specific mtDNA haplogroups, and LVDD stratified by sex.

## DISCUSSION

In a large sample consisting of men of European or African ancestry and women of African ancestry, we observed that specific mtDNA haplogroups were associated with significantly different susceptibility to LVDD among individuals with HIV. In initial analyses including both PWH and PWoH, no mtDNA haplogroup was significantly associated with LVDD in men or women. However, among non-Hispanic White men with HIV, European haplogroup H may confer protection against LVDD, while European haplogroup JT was associated with increased risk. The absence of significant associations in PWoH, together with the observation that HIV status modified the relationship between specific haplogroups and LVDD among women, suggests a potential physiological interplay between mitochondrial genetics and unique factors in PWH that may alter susceptibility to cardiac dysfunction. Additionally, prior D-drug exposure was associated with higher odds of LVDD in men, independent of mtDNA haplogroups. Together, these findings suggest a combination of genetic, infectious, and treatment-related mitochondrial stressors that may contribute to the elevated risk of cardiac dysfunction observed in PWH.

Previous studies that were the motivation for echocardiographic data collection in MACS and WIHS have reported on several cardiac parameters, including LVDD [29, 30]. In MACS, among 1195 men, LVDD defined by the CHART study criteria was more common in men with HIV (OR 1.43; 95% CI, 1.03–1.99), despite a slightly elevated prevalence among men without HIV (24.6% vs 23.1%) [30]. The WIHS study of 1654 women, applied the 2016 ASE criteria for LVDD and found no significant difference in the risk of LVDD between women with and without HIV (relative risk 1.69; 95% CI, .56–1.19) [29]. The stricter ASE 2016 definitions yielded lower LVDD prevalence estimates overall (6.5% in women without HIV vs 8.0% in women without HIV), whereas our study using the CHART study criteria observed markedly higher estimates (32.0% vs 28.2%).

While there is limited literature examining the association between mtDNA haplogroups and cardiac dysfunction, most existing studies focus on cardiomyopathies in PWoH. To our knowledge, no published studies have explored the relationship between African mtDNA haplogroups and cardiac dysfunction, which may reflect a gap in research or align with our observed lack of significant associations. In contrast to our findings on European haplogroup H and JT in men with HIV, haplogroup H in PWoH from Spanish and Danish populations have been described as more prevalent among individuals with idiopathic dilated, ischemic, and hypertrophic cardiomyopathy compared with healthy controls [24, 35, 36]. From the same studies, haplogroup J was less prevalent among persons with ischemic and hypertrophic cardiomyopathy [24, 36]. Although our results for men without HIV showed no statistical association with haplogroups H and JT, the direction of associations is consistent with these findings. The change in the direction of the association by HIV serostatus may suggest that HIV infection, or another exposure specific to PWH, modifies the association of mtDNA haplogroups and LVDD [37].

Additional research in PWH has revealed associations between mtDNA haplogroups and various metabolic conditions. In the MACS cohort, European haplogroup J was identified as a risk factor for gait speed decline, while haplogroup H was associated with weaker grip strength and frailty in men and women from the AIDS Clinical Trials Group A5322 (HAILO) [17, 38]. Emerging evidence also suggests that certain African and European mtDNA haplogroups alter the risk of DM in PWH [21, 39]. These variable associations across metabolic traits may reflect tissue-specific mitochondrial characteristics and divergent alterations in metabolic pathways underlying disease pathogenesis.

Although functional differences between mtDNA haplogroups have been demonstrated in vitro, how these variations translate to tissue-level phenotypes and clinical outcomes remains poorly understood [40, 41]. Mitochondria differ across tissues in morphology, abundance, and function, all of which are essential for maintaining organ-specific homeostasis [42]. For example, the heart exhibits the highest oxidative phosphorylation capacity and the greatest mtDNA content of any organ [43]. This feature may explain why African mtDNA haplogroups, which have been suggested to have enhanced oxidative phosphorylation efficiency [44], were not associated with LVDD in our study, yet have shown relevance in other metabolic conditions. It is possible that individuals with African haplogroups possess a greater capacity to buffer mitochondrial stress before impairment manifests as cardiac dysfunction. Future research should explore how cell-level mitochondrial traits translate into tissue-level susceptibility and clinical phenotypes.

While our ability to assess sex differences was limited by differences between the MACS and WIHS cohorts, LVDD prevalence was higher in women (29.4%) than in men (24.2%) regardless of HIV status, consistent with prior reports of greater LVDD and HFpEF prevalence in women [45, 46]. No African mtDNA haplogroup was significantly associated with LVDD, but HIV serostatus modified the associations of haplogroups L2 and L3 in women, an effect not seen in men. Although estimates for African mtDNA haplogroups in men were characterized by wide CIs, sex-related differences more broadly may reflect biological mechanisms. These include variation in sex chromosomes, hormone levels, and mitochondrial gene expression, with animal models also demonstrating sex-specific variations in mitochondrial function [47, 48].

Our findings on prior D-drug exposure align with earlier reports suggesting that the mitochondrial effects of these medications can persist long after they are discontinued [16]. We further demonstrated that previous D-drug exposure increased the risk of LVDD in men. The lack of significance among women may be due to a smaller proportion having prior D-drug exposure relative to men. Better characterizing the duration of D-Drug exposure, incorporating additional ART, and directly measuring mitochondrial function are critical to clarify the mechanisms by which HIV and its treatments contribute to cardiac dysfunction.

Since the introduction of ART, the cardiac dysfunction in PWH has evolved. Rates of left ventricular systolic dysfunction have declined, while LVDD has become increasingly prevalent [30, 31]. Chronic inflammation, even in the setting of viral suppression, has been proposed as a potential explanation for this shift [31]. Our findings support this hypothesis given the associations remained robust in sensitivity analyses accounting for plasma HIV RNA concentrations suggesting that the results were not driven by individuals with detectable viral loads. Another possible contributing factor to the change in cardiac dysfunction is the evolution of ART. While D-drugs are not prescribed in modern practice, other ART have also been implicated in mitochondrial dysfunction. For instance, abacavir–lamivudine and tenofovir disoproxil fumarate (TDF)–emtricitabine have been shown to reduce mtDNA content, with TDF–emtricitabine also further impairing the activity of mitochondrial respiratory chain complexes [49, 50]. These results emphasize the multifactorial nature of mitochondrial dysfunction in PWH and underscore the need to understand how genetic and pharmacologic factors interact to influence long-term cardiac outcomes to address the growing burden of cardiac dysfunction in this population.

This study has several limitations. First, the cross-sectional design limits our ability to assess temporal changes in cardiac function and precludes causal inference. Second, stratification by sex, HIV serostatus, and haplogroup reduced subgroup sizes, which may have limited our power to detect meaningful associations. Third, the generalizability of our findings may be affected by cohort-specific enrollment patterns. For instance, MACS participants who received echocardiograms tended to be younger, have a lower baseline cardiovascular risk, and were more likely to be PWH compared with all MACS participants, while WIHS participants with echocardiograms were more likely to be non-Hispanic Black, premenopausal or perimenopausal, and hypertensive compared with all WIHS participants. These differences between MACS and WIHS accounted for our sex-stratified analyses. Moreover, the limited granularity of information on cumulative D-drug exposure and other ART regimens prevented us from exploring dose-response effects and additional ART effects. Finally, given the exploratory nature of this analysis, we did not correct for multiple comparisons, which may have led to false positive associations. Nonetheless, to our knowledge, this study is the first to examine the relationship between mitochondrial haplogroups and cardiac dysfunction in PWH and offers critical insights into potential mechanisms underlying HIV-associated LVDD.

In summary, among non-Hispanic White men living with HIV, European mtDNA haplogroup H may offer protection against LVDD, while haplogroup JT may increase the risk. Prior exposure to D-drugs was also associated with increased odds of LVDD, suggesting the multitude of mitochondrial stressors in PWH contributing to cardiac dysfunction. These findings underscore the need for future research examining the interplay between HIV-related exposures, mitochondrial genetics, and time-varying risk factors. A more nuanced understanding of these interactions will provide insight into disease pathogenesis and could guide the development of targeted surveillance and therapeutic strategies for prevention and treatment of cardiac dysfunction.

## Supplementary Material

jiag090_Supplementary_Data

## Supplementary Data

Supplementary materials are available at *The Journal of Infectious Diseases* online (http://jid.oxfordjournals.org/). Supplementary materials consist of data provided by the author that are published to benefit the reader. The posted materials are not copyedited. The contents of all supplementary data are the sole responsibility of the authors. Questions or messages regarding errors should be addressed to the author.
