# Supplementary material for: Mitochondrial Haplogroups and Left Ventricular Diastolic Dysfunction in People Living With and Without HIV
Source: J Infect Dis. 2026 Feb 11;233(6):e1423–32. doi: 10.1093/infdis/jiag090 (PMC13271400; doi:10.1093/infdis/jiag090)
Supplement: jiag090_Supplementary_Data [file jiag090_supplementary_data.docx]

**Supplementary Materials:**

**Supplementary Methods:**

Study Population:

The WIHS sites included Bronx, NY; Brooklyn, NY; Chicago, IL; Washington, DC; Chapel Hill, NC; Atlanta, GA; Miami, FL; Birmingham, AL; Jackson, MS; San Francisco, CA and MACS site included Baltimore/Washington DC; Chicago, IL; Pittsburgh, PA; Columbus, OH; and Los Angeles, CA.

The WIHS-wide echocardiographic study was conducted between 2014 and 2019 (n=1,654 women). The echocardiographic study was piloted in Bronx and Brooklyn sites from 2014 to 2016 and then received funding for a larger study from 2016 to 2019. The MACS-wide echocardiographic study was conducted from October 2017 to January 2019 (n=1,195 men). Of note, each participant included in this study underwent a single echocardiogram.

Assessment of Left Ventricular Diastolic Dysfunction:

The CHART study criteria characterizes LVDD by 1) preserved ejection fraction (LV ejection fraction $\geq$50%, 2) evidence of impaired LV relaxation (septal e’ <7 cm/s or lateral e’<10 cm/s), and 3) evidence of chronically elevated LV filling pressure (LA maximum volume index >28 mL/m^2^), LV hypertrophy (LV mass index >115 g/m^2^), or concentric LV remodeling (relative wall thickness >0.42).

**Supplementary Table 1:** Unadjusted and Adjusted Associations between all African or European Haplogroups and Left Ventricular Diastolic Dysfunction for Males and Females, Included p-values for Interaction between HIV Serostatus and mtDNA Haplogroups

|  | All | | HIV+ | | | | | | HIV- | | | | | |
| --- | --- | --- | --- | --- | --- | --- | --- | --- | --- | --- | --- | --- | --- | --- |
| Haplogroup | Univariable | | Model 1 | | Model 2 | | Model 3 | | Model 1 | | Model 2 | | Model 3 | |
|  | OR  (95% CI) | P-value | OR  (95% CI) | P-value | OR  (95% CI) | P-value | OR  (95% CI) | P-value | OR  (95% CI) | P-value | OR  (95% CI) | P-value | OR  (95% CI) | P-value |
| **MALE** |  |  |  |  |  |  |  |  |  |  |  |  |  |  |
| L0L1 vs non- L0L1  p_int_=0.48 | 0.798  (0.379, 1.582) | 0.532 | 1.024  (0.389, 2.501) | 0.959 | 1.074  (0.378, 2.846) | 0.889 | 1.065  (0.372, 2.848) | 0.903 | 0.478  (0.108, 1.777) | 0.292 | 0.414  (0.050, 2.367) | 0.358 | 0.313  (0.029, 2.226) | 0.280 |
| L2 vs non-L2  p_int_=0.55 | 0.762  (0.408, 1.381) | 0.380 | 0.751  (0.322, 1.682) | 0.495 | 0.895  (0.350, 2.218) | 0.812 | 1.012  (0.378, 2.633) | 0.981 | 0.737  (0.212, 2.379) | 0.616 | 0.740  (0.179, 2.886) | 0.665 | 0.789  (0.163, 3.578) | 0.758 |
| L3 vs non-L3  p_int_=0.96 | 1.532  (0.871, 2.689) | 0.137 | 1.328  (0.612, 2.870) | 0.470 | 1.097  (0.462,  2.578) | 0.832 | 1.006  (0.415, 2.405) | 0.988 | 2.019  (0.655, 6.424) | 0.223 | 1.692  (0.453, 6.711) | 0.437 | 1.900  (0.433, 9.157) | 0.400 |
| H vs non-H  p_int_=0.90 | 0.771  (0.52, 1.134) | 0.189 | 0.517  (0.283, 0.931) | 0.029 | 0.464  (0.247,  0.851) | 0.014 | 0.496  (0.260, 0.926) | 0.030 | 1.034  (0.578, 1.836) | 0.909 | 0.923  (0.507, 1.663) | 0.791 | 1.020  (0.554, 1.866) | 0.948 |
| UK vs non-UK  p_int_=0.75 | 1.071  (0.682, 1.655) | 0.760 | 1.271  (0.607, 2.584) | 0.514 | 1.307  (0.608, 2.733) | 0.482 | 1.190  (0.538, 2.555) | 0.660 | 1.071  (0.564, 1.985) | 0.830 | 1.233  (0.634, 2.356) | 0.530 | 1.104  (0.556, 2.147) | 0.772 |
| JT vs non-JT  p_int_=0.18 | 1.293  (0.815, 2.019) | 0.266 | 1.960  (0.977, 3.890) | 0.055 | 2.106  (1.023, 4.310) | 0.041 | 2.093  (1.000, 4.363) | 0.048 | 0.790  (0.383, 1.554) | 0.506 | 0.769  (0.364, 1.550) | 0.475 | 0.776  (0.364, 1.583) | 0.496 |
| **FEMALE** |  |  |  |  |  |  |  |  |  |  |  |  |  |  |
| L0L1 vs non- L0L1  p_int_=0.089 | 1.026  (0.727, 1.435) | 0.883 | 0.998  (0.637, 1.547) | 0.992 | 1.042  (0.659, 1.633) | 0.857 | 0.968  (0.606, 1.531) | 0.889 | 1.039 (0.497, 2.132) | 0.918 | 0.981  (0.450, 2.102) | 0.961 | 0.897  (0.397, 1.985) | 0.791 |
| L2 vs non-L2  p_int_=0.036 | 0.910  (0.658, 1.250) | 0.565 | 0.831  (0.533, 1.279) | 0.405 | 0.811  (0.516, 1.262) | 0.359 | 0.870  (0.547, 1.370) | 0.551 | 1.106  (0.575, 2.107) | 0.761 | 1.217  (0.611, 2.415) | 0.575 | 1.255  (0.613, 2.566) | 0.533 |
| L3 vs non-L3  p_int_=0.045 | 1.152  (0.861, 1.540) | 0.340 | 1.140  (0.775, 1.673) | 0.504 | 1.126  (0.759, 1.668) | 0.553 | 1.096  (0.732, 1.637) | 0.656 | 1.317  (0.702, 2.473) | 0.390 | 1.331  (0.679, 2.621) | 0.404 | 1.359  (0.675, 2.753) | 0.391 |

Abbreviations: CI, confidence interval; HIV, human immunodeficiency virus; OR, odds ratio; p_int_, p-value for interaction between HIV serostatus and mitochondrial DNA Haplogroups; -, seronegative; +, seropositive

Model 1 adjusted for two principal components of nuclear genetic ancestry, age, and site of enrollment.

Model 2 further adjusted for BMI, educational attainment, alcohol use, HCV serostatus, smoking, and history of non-injection or injection drug use.

Model 3 further adjusted for HTN, DM, dyslipidemia, and eGFR

**Supplementary Table 2:** Final Adjusted Model (Model 3) with Addition of HIV-specific Covariates for Associations Between all African or European Haplogroups and Left Ventricular Diastolic Dysfunction for Males and Females with HIV

| Haplogroup | Model 3 + HIV-Specific Covariates | |
| --- | --- | --- |
|  | OR  (95% CI) | P-value |
| **MALE** |  |  |
| L0L1 vs non- L0L1 | 1.100  (0.350, 3.230 | 0.864 |
| L2 vs non-L2 | 1.035  (0.384, 2.730) | 0.945 |
| L3 vs non-L3 | 0.912  (0.349, 2.330) | 0.848 |
| H vs non-H | 0.439  (0.225, 0.835) | 0.013 |
| UK vs non-UK | 1.291  (0.578, 2.809) | 0.525 |
| JT vs non-JT | 2.263  (1.055, 4.868) | 0.035 |
| **FEMALE** |  |  |
| L0L1 vs non- L0L1 | 1.009  (0.625, 1.612) | 0.971 |
| L2 vs non-L2 | 0.828  (0.514, 1.318) | 0.430 |
| L3 vs non-L3 | 1.143  (0.758, 1.720) | 0.522 |

Abbreviations: CI, confidence interval; HIV, human immunodeficiency virus; OR, odds ratio; -, seronegative; +, seropositive

Model 3 further adjusted for CD4/CD8 ratio, history of AIDS, 3-year average viremia, and nadir CD4 count

**Supplementary Table 3:**  Final Adjusted Model (Model 3) for Associations Between Statistically Significant European Haplogroups from Supplementary Table 1 and Left Ventricular Diastolic Dysfunction for Males with HIV and HIV RNA $\leq$ 20 copies /mL

|  | Model 3 Limited to Males with HIV and HIV RNA $\leq$ 20 copies /mL | |
| --- | --- | --- |
|  | OR  (95% CI) | P-value |
| H vs non-H  (n = 118) | 0.521  (0.255, 1.042) | 0.068 |
| JT vs non-JT  (n = 46) | 2.911  (1.271, 6.761) | 0.012 |

Abbreviations: CI, confidence interval; OR, odds ratio

Model 3 adjusted for two principal components of nuclear genetic ancestry, age, and site of enrollment, BMI, educational attainment, alcohol use, HCV serostatus, smoking, history of non-injection or injection drug use, HTN, DM, dyslipidemia, and eGFR

**Supplementary Table 4:** Unadjusted and Adjusted Odds Ratio of Left Ventricular Diastolic Dysfunction for People with HIV and Previous D-drug Exposure Compared to People with HIV without D-drug Exposure

|  | Univariable  OR (95% CI) | Controlled for HIV disease severity^a^  OR (95% CI) |
| --- | --- | --- |
| Overall | 1.37 (1.04, 1.79) | 1.31 (0.99, 1.74) |
| Female | 1.11 (0.77, 1.58) | 1.09 (0.74, 1.58) |
| Male | 2.09 (1.25, 3.25) | 1.94 (1.21, 3.13) |

Abbreviations: CI, confidence interval; HIV, human immunodeficiency virus; OR, odds ratio

^a^Adjusted for CD4/CD8 ratio, history of AIDS, 3-year average viremia, and nadir CD4 count

**Supplementary Table 5:** Adjusted Odds Ratios for Left Ventricular Diastolic Dysfunction among HIV Positive Females Grouped by D-drug Exposure and Specific Mitochondrial Haplogroups

| Group | Comparison | Model 3 |
| --- | --- | --- |
|  |  | OR (95% CI) |
| African L2 | D-drug (+) vs. D-drug (-) | 22.501  (2.998, 252.475) |
| African non-L2 | D-drug (+) vs. D-drug (-) | 0.838  (0.442, 1.585) |
| African L3 | D-drug (+) vs. D-drug (-) | 0.929  (0.373, 2.305) |
| African non-L3 | D-drug (+) vs. D-drug (-) | 1.181  (0.538, 2.602) |
| D-drug (-) | African L2 vs. non-L2 | 0.699  (0.395, 1.210) |
| D-drug (+) | African L2 vs. non-L2 | 1.120  (0.370, 3.361) |
| D-drug (-) | African L3 vs. non-L3 | 1.142  (0.702, 1.855) |
| D-drug (+) | African L3 vs. non-L3 | 1.394  (0.567, 3.449) |

Abbreviations: CI, confidence interval; OR, odds ratio

Model 3 adjusted for two principal components of nuclear genetic ancestry, age, and site of enrollment, BMI, educational attainment, alcohol use, HCV serostatus, smoking, history of non-injection or injection drug use, HTN, DM, dyslipidemia, and eGFR. Additionally adjusted for CD4/CD8 ratio, history of clinical AIDS, 3-year average HIV viremia, and nadir CD4 count.

**Supplementary Table 6:** Adjusted Odds Ratios for Left Ventricular Diastolic Dysfunction among HIV Positive Males Grouped by D-drug Exposure and Specific Mitochondrial Haplogroups

| Group | Comparison | Model 3 | |
| --- | --- | --- | --- |
|  |  | OR (95% CI) | p-value |
| European H | D-drug (+) vs. D-drug (-) | 0.999 (0.225, 4.264) | 0.99 |
| European non-H | D-drug (+) vs. D-drug (-) | 1.515 (0.521, 4.506) | 0.45 |
| European JT | D-drug (+) vs. D-drug (-) | - | - |
| European non-JT | D-drug (+) vs. D-drug (-) | 1.589 (0.606, 4.236) | 0.35 |
| African L2 | D-drug (+) vs. D-drug (-) | - | - |
| African non-L2 | D-drug (+) vs. D-drug (-) | 0.525 (0.125, 1.976) | 035 |
| African L3 | D-drug (+) vs. D-drug (-) | 0.156 (0.010, 1.426) | 0.13 |
| African non-L3 | D-drug (+) vs. D-drug (-) | 2.029 (0.390, 11.465) | 0.40 |
| D-drug (-) | European H vs. non-H | 0.254 (0.053, 1.033) | 0.065 |
| D-drug (+) | European H vs. non-H | 0.348 (0.132, 0.862) | 0.026 |
| D-drug (-) | European JT vs. non-JT | 9.280 (1.653, 69.459) | 0.017 |
| D-drug (+) | European JT vs. non-JT | 1.560 (0.505, 4.798) | 0.47 |
| D-drug (-) | African L2 vs. non-L2 | 0.140 (0.006, 1.817) | 0.16 |
| D-drug (+) | African L2 vs. non-L2 | 2.445 (0.506, 13.920) | 0.28 |
| D-drug (-) | African L3 vs. non-L3 | 13.489 (1.196, 420.628) | 0.071 |
| D-drug (+) | African L3 vs. non-L3 | 0.181 (0.024, 0.984) | 0.064 |

Abbreviations: CI, confidence interval; OR, odds ratio

Model 3 adjusted for two principal components of nuclear genetic ancestry, age, and site of enrollment, BMI, educational attainment, alcohol use, HCV serostatus, smoking, history of non-injection or injection drug use, HTN, DM, dyslipidemia, and eGFR. Additionally adjusted for CD4/CD8 ratio, history of clinical AIDS, 3-year average HIV viremia, and nadir CD4 count.
